# Association of torque teno virus in periodontitis: a systematic review, meta-analysis, and trial sequential analysis

**DOI:** 10.3389/froh.2025.1714677

**Published:** 2025-11-20

**Authors:** Luis Chauca-Bajaña, Mario Pérez-Sayáns, Alejandro Ismael Lorenzo-Pouso, Byron Velasquez-Ron, Rafael Xavier Erazo Vaca, Rolando Fabricio Dau Villafuerte, Veronica Natalia Maroto Hidalgo, Jossue Tarquino Narváez Guerrero, César Humberto Palacios Jurado, Mercedes Treviño Castellano, Andrea Ordóñez Balladares

**Affiliations:** 1 Periodontics and Implantology Oral Research, College Dentistry, Ecuador, Faculty of Medicine and Dentistry, University of Guayaquil, Guayas, Ecuador; 2 Oral Medicine, Oral Surgery and Implantology Unit (MedOralRes), Faculty of Medicine and Dentistry Universidade de Santiago de Compostela, Santiago de Compostela, Spain; 3 Instituto de Investigación Sanitaria de Santiago (IDIS), ORALRES Group Santiago de Compostela, Santiago de Compostela, Spain; 4 Instituto de los Materiales de Santiago de Compostela (iMATUS), Santiago de Compostela, Spain; 5 Carrera de Odontología, Universidad de Las Américas (UDLA), Department Prosthesis Research, Quito, Ecuador; 6 Oral Rehabilitation, College Dentistry, Ecuador, Faculty of Medicine and Dentistry, University of Guayaquil, Guayas, Ecuador; 7 Endodontics, College Dentistry, Ecuador, Faculty of Medicine and Dentistry, University of Guayaquil, Guayas, Ecuador; 8 Servicio de Microbiología, Complejo Hospitalario Universitario de Santiago de Compostela, Santiago de Compostela, Spain; 9 College Dentistry, University Bolivariana del Ecuador, Durán, Ecuador

**Keywords:** torque teno virus, periodontitis, virome, molecular diagnostics, meta-analysis

## Abstract

**Background:**

Periodontitis, affecting 38.5% of adults globally with moderate-to-severe forms, represents a multifactorial inflammatory disease traditionally attributed to bacterial pathogens. Emerging evidence implicates viral cofactors, particularly Torque Teno virus (TTV), a ubiquitous anellovirus with 30%–95% prevalence in healthy populations.

**Objective:**

To evaluate TTV prevalence in periodontitis patients vs. controls through systematic review and meta-analysis.

**Methods:**

Following PRISMA guidelines, we searched multiple databases (2000–2024) for observational studies reporting TTV detection in oral samples using molecular techniques. Random-effects meta-analysis calculated pooled relative risk (RR) with 95% confidence intervals. Trial Sequential Analysis assessed evidence sufficiency.

**Results:**

Four studies encompassed 583 participants (300 periodontitis, 283 controls). Meta-analysis revealed significant TTV-periodontitis association (RR = 1.67; 95%CI: 1.28–2.17; *p* < 0.001), indicating 67% increased TTV likelihood in periodontitis patients. Heterogeneity was minimal (*I*^2^ = 0%) with no publication bias detected. Trial Sequential Analysis showed current evidence represents only 31.6% of required information size (1,847 participants), suggesting preliminary findings requiring validation.

**Conclusion:**

Despite consistent TTV-periodontitis association across studies, evidence remains insufficient for definitive conclusions. Larger prospective investigations using standardized diagnostic criteria are essential to establish causality and clinical significance.

**Systematic Review Registration:**

PROSPERO CRD420251127439.

## 1 Introduction

Periodontal disease is a chronic multifactorial condition that leads to the destruction of the tooth-supporting tissues, including bone, cementum, and the periodontal ligament (1, 2). It is estimated that nearly 50% of the global adult population presents some form of periodontal disease, with approximately 38.5% suffering from moderate to severe periodontitis (stages III and IV) (3, 4). Bacteria such as Porphyromonas gingivalis, Tannerella forsythia, Aggregatibacter actinomycetemcomitans, and Treponema denticola are considered the primary etiological agents involved in the development of periodontal disease (5–7). However, since the mid-1990s, viruses have also been implicated in the pathogenesis of periodontal diseases, suggesting a potential role as cofactors that may influence the host immune response and the severity of tissue damage (8, 9). Previous evidence has demonstrated that periodontal lesions can harbor millions of genomic copies of viruses such as herpesvirus, HPV, human T-cell lymphotropic virus type I (HTLV-I) (9), hepatitis B (8) and C viruses (10), human immunodeficiency virus (HIV), and Torque Teno virus (TTV) (11), supporting the hypothesis of a viral contribution to the pathogenesis of periodontal disease.

Torque Teno virus (TTV) was first identified in 1997 as a novel DNA virus potentially associated with cases of post-transfusion hepatitis not attributable to conventional hepatitis viruses (12). It belongs to the Anelloviridae family, also referred to as Alphatorquevirus (13). TTV is now recognized as a ubiquitously distributed virus with high prevalence rates even among asymptomatic individuals, ranging from 30% to 95% (14–16). Moreover, TTV DNA has been detected in a wide range of biological samples, including serum, bone marrow, lung, liver, and lymph nodes (17). In previous investigations, a novel anellovirus species, designated Torque Teno Mini Virus 222 (TTMV-222), was identified in gingival tissue from patients with periodontitis (18). Rotundo et al. (11) further reported a higher prevalence of TTV in individuals with chronic periodontitis compared with healthy controls in an Italian population.

Despite these preliminary findings linking TTV to periodontal lesions, the evidence is still limited and scattered. In this review, we bring together what is known about how often TTV is found in people with periodontitis and consider what that might mean clinically. Because *Torque teno virus* (TTV) and *Torque teno mini virus* (TTMV) are closely related anelloviruses and the main difference between them is simply genome size, with no proven biological or clinical implications in periodontal tissues we analyzed them together in the primary synthesis and, when available, noted whether a study targeted TTV or TTMV.

## 2 Materials and methods

### 2.1 Protocol and registration

**Figure 1:**
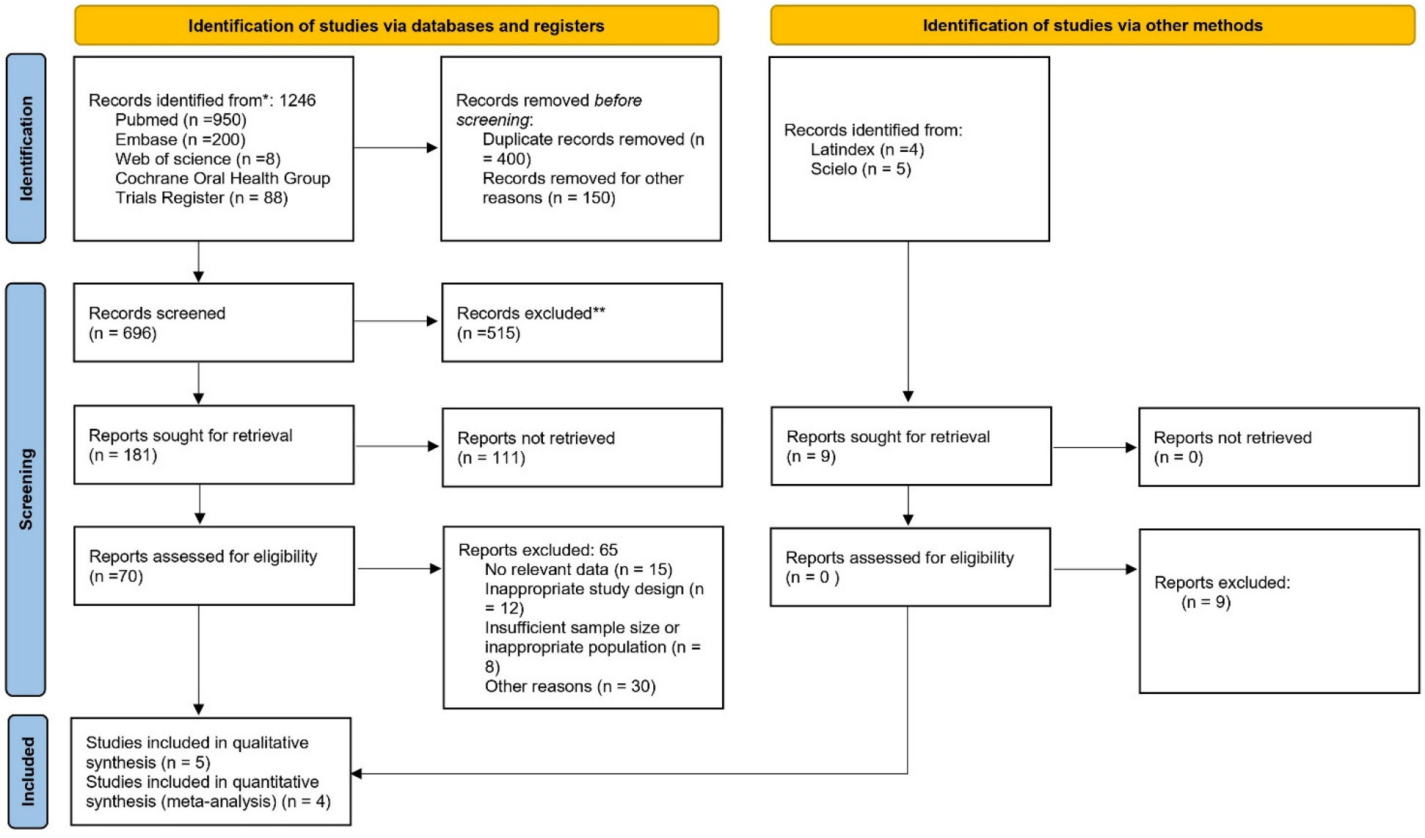
Flowchart of selected studies.

A study protocol was developed *a priori* to guide the search and data retrieval process, following the PRISMA guidelines (19) (Figure 1). The protocol was prospectively registered in PROSPERO (registration ID: CDR420251127439) to minimize the risk of bias and ensure methodological transparency.

### 2.2 PICO question

What is the prevalence of Torque Teno Virus (TTV) in individuals with periodontitis compared with healthy individuals?

• **P (Population):** Adults with periodontal disease
• **I (Intervention/Exposure):** Presence of Torque Teno Virus (TTV)
• **C (Comparison):** Adults without periodontal disease (when comparative data are available)
• **O (Outcome):** Prevalence of TTV detected in oral samples (saliva, gingival crevicular fluid, gingival tissue)

### 2.3 Search strategy and database screening

The Rayyan QCRI platform (Qatar Computing Research Institute, Doha, Qatar) was used to facilitate the identification and selection of eligible articles. The search strategy involved a comprehensive review across multiple databases: MEDLINE (PubMed), EMBASE (OVID), Web of Science, Scopus, the Cochrane Library, ClinicalTrials.gov, and the five regional bibliographic databases of the WHO (AIM, LILACS, IMEMR, IMSEAR, WPRIM), as well as the Conference Proceedings Citation Index. A tailored combination of keywords and MeSH terms was constructed and refined for each database to maximize search sensitivity. The core search strategy included the following terms: *Torque Teno Virus*, TTV, anellovirus, periodontitis, periodontal disease, gingival crevicular fluid, saliva, PCR, prevalence; Boolean operators AND/OR were applied as appropriate to combine terms.

### 2.4 Eligibility criteria

#### 2.4.1 Inclusion criteria

• Observational studies (cross-sectional, case-control, cohort) reporting:
  ▪ Prevalence of TTV in individuals with periodontal disease
  ▪ Detection of TTV in oral samples (saliva, gingival crevicular fluid, subgingival plaque)
• Human studies including adults (≥18 years)
• Articles published in English
• Studies published from the year 2000 onwards

#### 2.4.2 Exclusion criteria

• Animal or *in vitro* studies
• Reviews, editorials, letters to the editor
• Studies not reporting quantifiable data on TTV prevalence
• Studies in which data from healthy and periodontitis patients could not be analyzed separately

### 2.5 Study selection and data extraction

Two reviewers (L.CH and B.V.R) independently performed study selection and data extraction using a pre-designed, standardized data extraction form developed for this review. Discrepancies between reviewers were resolved through discussion, and when necessary, a third reviewer (M.P.S), blinded to the primary study hypothesis, was consulted.

The following data were extracted from each included study: first author, year of publication, country, study design, total number of participants, periodontal diagnosis, type of oral sample (saliva, gingival crevicular fluid, subgingival plaque), Torque Teno Virus detection method (conventional PCR, qPCR, etc.), target regions or genes, complementary techniques, and study conclusions.

The selection process began with screening of titles and abstracts of all records identified through the database searches. Potentially eligible studies were then assessed at full text to determine inclusion. In cases where essential data were missing or unclear, attempts were made to contact the corresponding authors to obtain clarifications or additional relevant information for the analysis. The detailed characteristics of the included studies are presented in Table 1. Five studies fulfilled the inclusion criteria [Zhang et al. (18); Zhang et al. (20); Rotundo et al. (11); Yu et al. (22); Priyanka et al. (21)]. However, Priyanka et al. (21) did not provide comparative data between periodontitis and control groups, and was therefore included in the qualitative synthesis but excluded from the quantitative meta-analysis due to the lack of extractable comparative data.

**Table 1:** Characteristics of included studies on torque teno virus detection in patients with periodontitis.

Author	Country	Study design	Sample size	Periodontal diagnosis	Oral sample	TTV detection method	Main finding
Zhang et al. (20)	China	Case-control	160	Chronic periodontitis (BOP, PD, CAL, bone loss); controls healthy	Gingival epithelium and sulcus-oriented connective tissue	Viral metagenomics (Illumina MiSeq) + hemi-nested PCR	Novel TTMV more prevalent in periodontitis patients vs. healthy subjects
Zhang et al. (18)	China	Cross-sectional	300	Severe periodontitis (CAL ≥3 mm, PD ≥6 mm, BOP, alveolar bone loss)	Gingival epithelium and connective tissue	Viral metagenomics + nested PCR	TTMV-222 detected more in periodontitis patients than controls (*p* = 0.032)
Priyanka et al. (21)	India	Cross-sectional	30	CAL >5 mm, PPD >5 mm, radiographic bone loss	Subgingival plaque	PCR (primers T801/T935)	TTV detected in subgingival and atherosclerotic plaque, suggesting systemic involvement
Rotundo et al. (11)	Italy	Cross-sectional comparative	21	Moderate periodontitis (CAL ≥6 mm, PD ≥5 mm)	Saliva and gingival biopsy	RT-PCR (TaqMan)	TTV in gingival tissue associated with periodontitis (*P* = 0.0351); no association in saliva
Yu et al. (22)	China	Cross-sectional	159	Chronic and aggressive periodontitis	Gingival biopsy	Nested-PCR and RT-PCR	TTV significantly associated with periodontitis; higher viral load with EBV co-infection

BOP, Bleeding on Probing; PD, Probing Depth; CAL, Clinical Attachment Loss; PPD, Periodontal Probing Depth; TTV, Torque Teno Virus; TTMV, Torque Teno Mini Virus; PCR, Polymerase Chain Reaction; RT-PCR, Real-Time Polymerase Chain Reaction.

Given that TTV and TTMV belong to the same *Anelloviridae* family and share similar replication and tissue distribution characteristics, studies detecting either subtype were included in the pooled analysis. Subtype differences (TTV vs. TTMV) were recorded and described qualitatively, as available data were insufficient for a formal subgroup meta-analysis.

### 2.6 Risk of bias assessment

Two authors (L.CH.B, B.V.R) independently assessed the included studies, using all verification items adapted for observational studies. The risk of bias was classified as “low,” “unclear,” or “high.” Case-control and cross-sectional studies were analyzed across five domains: (1) Definition of groups and selection bias—it was evaluated whether the case groups (patients with periodontitis) and controls (periodontally healthy individuals) were clearly defined and appropriately selected; (2) Comparability of groups—it was assessed whether baseline characteristics such as age, sex, and clinical parameters (PD, CAL, PLI, GI) were comparable between groups; (3) Completeness of outcome data—it was analyzed whether studies reported the total number of participants, TTV detection rates, methods used, and periodontal clinical measures; (4) Selective reporting—it was evaluated whether studies reported all pre-specified outcomes related to TTV prevalence and periodontal status without omitting relevant findings; and (5) Other potential sources of bias—confounding variables such as systemic diseases, previous periodontal treatments, or other viral co-infections were considered. Discrepancies were resolved by consensus. The overall assessment of risk of bias for the included studies is summarized in Table 2.

**Table 2:** Risk of bias assessment of included studies.

Study	Selection bias		Performance bias	Detection bias	Attrition bias	Reporting bias	Other biases
Author	Sequence Generation	Allocation concealment	Blinding of participants and personnel	Blinding of outcome assessors	Incomplete outcome data	Selective reporting of results	Other sources of bias
Zhang et al. (20)	Yes	Unclear	Yes	Yes	No	No	No
Zhang et al. (18)	Yes	Unclear	Unclear	Yes	No	No	No
Priyanka et al. (21)	Yes	Unclear	Unclear	Yes	No	No	No
Rotundo et al. (11)	Yes	Unclear	Unclear	Yes	No	No	No
Yu et al. (22)	Yes	Unclear	Yes	Yes	No	No	No
		Positive (good) indicator		Unclear		Negative (bad) indicator	

### 2.7 Statistical analysis

Statistical analysis was conducted using Review Manager 5.4.1 and R software 4.2.0. Pooled relative risks with 95% confidence intervals were estimated using random-effects models with the Mantel-Haenszel method. Heterogeneity was assessed using Higgins' *I*^2^ statistic, with *I*^2^ < 30% considered low heterogeneity. Publication bias was evaluated through funnel plots and Egger's regression test. Trial Sequential Analysis (TSA) was used to assess evidence sufficiency and control random errors in cumulative meta-analysis. Analyses were run in TSA software (v0.9.5.10 Beta, Copenhagen Trial Unit) under a random-effects model, using default parameters: two-sided *α* = 0.05, *β* = 0.20 (80% power), and O'Brien–Fleming–type monitoring boundaries. The diversity-adjusted required information size (DARIS) was estimated to judge whether the cumulative Z-curve crossed trial sequential boundaries (conclusive) or indicated the need for additional studies (23, 24).

## 3 Results

Five observational studies met inclusion criteria [Zhang et al. (18); Zhang et al. (20); Rotundo et al. (11); Yu et al. (22); Priyanka et al. (21)]. Among these, four provided comparative data suitable for meta-analysis, encompassing 583 participants (300 with chronic periodontitis and 283 healthy controls). The study by Priyanka et al. (21) was analyzed qualitatively because it did not include a control group and therefore lacked extractable risk estimates. Studies were conducted between 2004 and 2020, spanning the transition period of periodontal disease classification systems. All studies investigated chronic generalized periodontitis using pre-2017 AAP classification criteria, ensuring diagnostic homogeneity.

**Figure 2:**
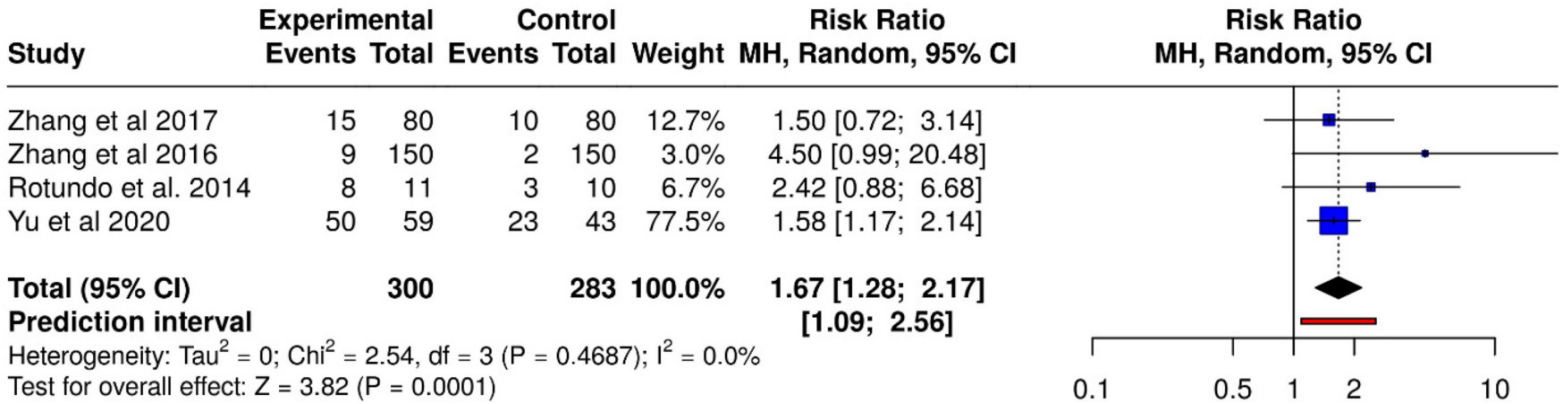
Forest plot of relative risk (RR) for TTV detection in periodontitis patients vs. healthy controls using random-effects model.

Meta-analysis using random-effects models demonstrated a significant association between TTV presence and chronic periodontitis (RR = 1.67; 95% CI: 1.28–2.17; *p* = 0.00013), indicating that individuals with periodontitis have a 67% greater likelihood of harboring TTV compared to healthy controls. The general effect test was statistically significant (*Z* = 3.82). Heterogeneity was minimal (Tau^2^ = 0; Chi^2^ = 2.54; df = 3; *p* = 0.47; *I*^2^ = 0%), suggesting consistency and robustness in findings across studies despite variations in detection methodologies and sample types (Figure 2).

**Figure 3:**
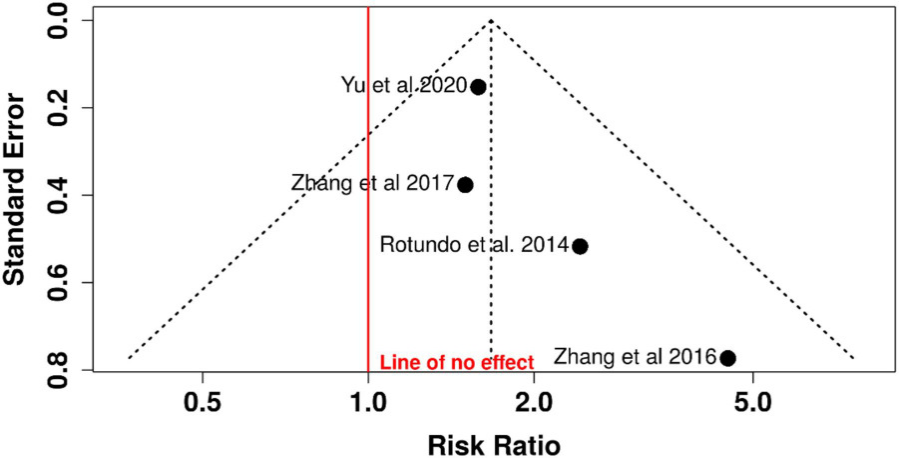
Funnel plot for relative risk analysis: assessment of publication bias in TTV association with periodontitis.

Funnel plot analysis revealed symmetrical distribution without visual evidence of publication bias. Egger's regression test confirmed this observation (intercept = 1.13; 95% CI: −0.01–2.27; *t* = 1.937; *p* = 0.192), with the non-significant *p*-value indicating absence of statistically significant asymmetry or small-study effects (Figure 3).

**Figure 4:**
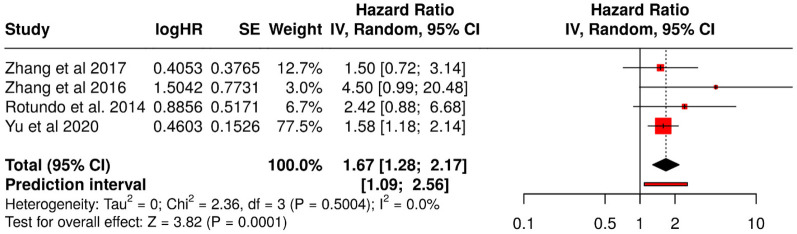
Forest plot of the hazard ratio (HR) for TTV prevalence using the inverse variance (IV) random-effects model with 95% prediction interval.

Four observational studies were pooled in a random-effects meta-analysis (inverse variance method; Figure 4). The combined hazard ratio was 1.67 (95% CI: 1.28–2.17; *p* = 0.0001), indicating a significantly higher risk of TTV detection in individuals with periodontitis compared with healthy controls. The 95% prediction interval (1.09–2.56) suggests that this association is likely to persist in future studies. Heterogeneity was negligible (*I*^2^ = 0%; Tau^2^ = 0; *χ*^2^ = 2.36, df = 3; *p* = 0.50) (Figure 4).

**Figure 5:**
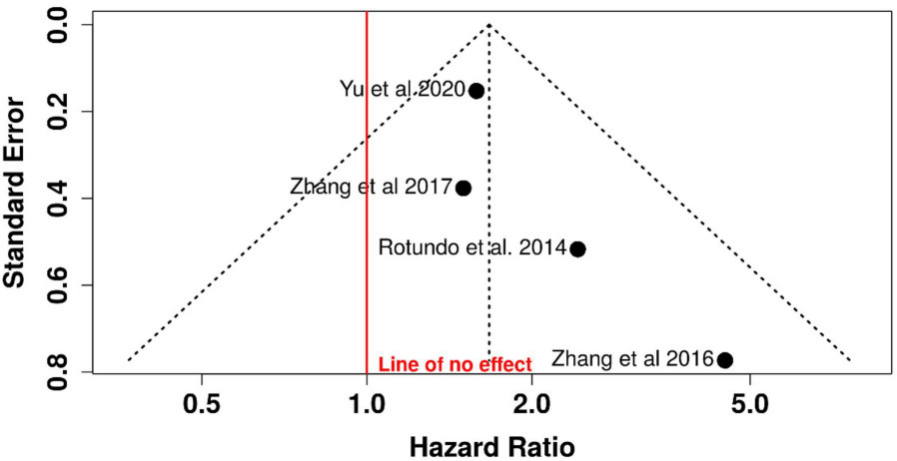
Funnel plot for risk ratio analysis: evaluation of small-study effects and publication bias in TTV risk ratio analysis.

The funnel plot (Figure 5) showed a symmetric distribution of studies around the estimated effect, with no visual evidence of asymmetry. Egger's test confirmed this observation (intercept = 1.13; 95% CI: −0.01–2.27; *t* = 1.935; *p* = 0.193), indicating no statistically significant evidence of publication bias.

The absence of publication bias suggests that the meta-analysis results are unlikely to be influenced by the selective omission of negative or non-significant studies, thereby reinforcing the external validity of the conclusions.

**Figure 6:**
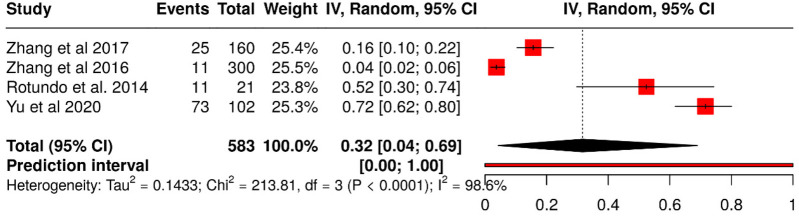
Forest plot of TTV proportion analysis using freeman-tukey double transformation for variance stabilization.

The meta-analysis included 583 participants from four observational studies evaluating the association between Torque Teno Virus (TTV) infection and periodontitis (Figure 6). A random-effects model was applied using the inverse variance method with the Freeman–Tukey double arcsine transformation to stabilize variances and enhance comparability across studies.

The pooled hazard ratio was 1.67 (95% CI: 1.28–2.18; *t* = 3.82; *p* = 0.00013), indicating that patients with periodontitis had a 67% higher likelihood of TTV detection compared with healthy controls. However, substantial heterogeneity was observed (Tau^2^ = 0.1433; *χ*^2^ = 213.81; *p* < 0.0001; *I*^2^ = 98.6%), suggesting that variability in effect sizes is largely attributable to methodological and population differences across studies rather than chance.

**Figure 7:**
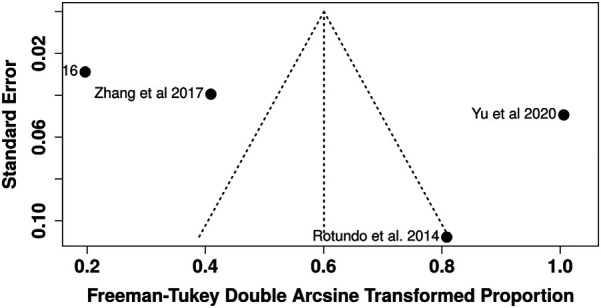
Funnel plot for TTV proportion analysis: assessment of asymmetry and publication bias.

The funnel plot (Figure 7) showed no visual evidence of asymmetry. Egger's test also did not support the presence of publication bias (intercept = 13.08; 95% CI: −6.57 to 32.73; *t* = 1.305; *p* = 0.322). These findings suggest that the results are unlikely to be influenced by the systematic omission of studies with negative or non-significant outcomes.

However, given the high level of heterogeneity detected, it is possible that the apparent symmetry of the funnel plot may be masking sources of bias related to methodological variability across studies rather than the absence of unpublished data.

**Figure 8:**
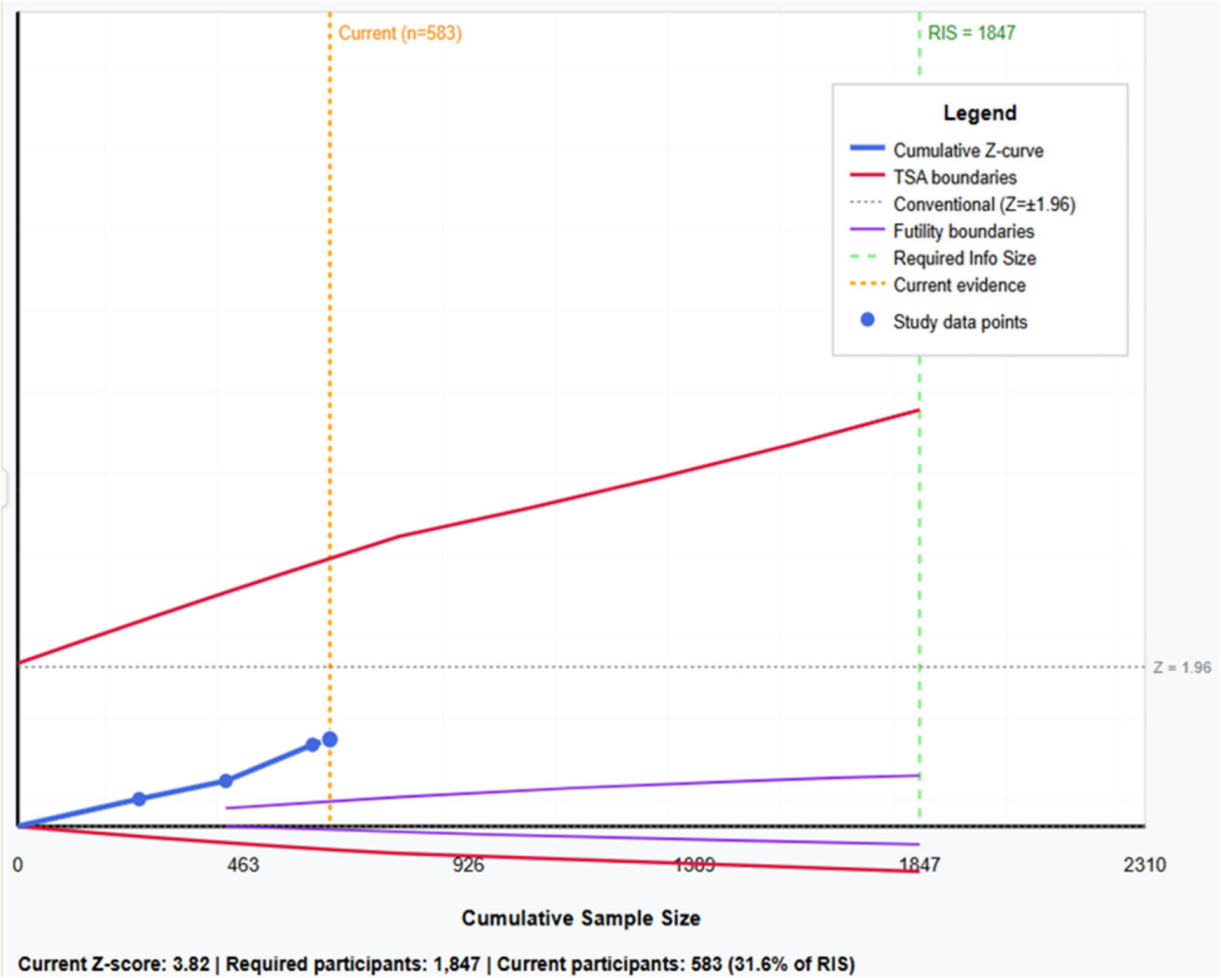
Trial sequential analysis: cumulative Z-curve with trial sequential monitoring boundaries, conventional statistical boundaries, and required information size (RIS = 1,847 participants) for TTV-periodontitis association.

Trial Sequential Analysis revealed important limitations in current evidence sufficiency. While conventional meta-analysis demonstrated statistical significance, TSA showed that the cumulative sample size (*n* = 583) represents only 31.6% of the required information size (1,847 participants). The cumulative Z-curve crossed conventional significance boundaries but failed to cross trial sequential monitoring boundaries, suggesting that observed statistical significance may be due to random error rather than true association. The analysis indicates that approximately 1,264 additional participants across well-designed studies would be needed to provide conclusive evidence (Figure 8).

## 4 Discussion

Periodontitis is one of the most prevalent periodontal diseases worldwide (3, 25). Traditionally, its etiology has been attributed to periodontopathogenic bacteria; however, the heterogeneity of observed clinical manifestations suggests that bacterial presence alone does not fully explain disease progression (26). Current evidence supports a multifactorial etiology, in which the interaction between the microbiota, host response, and chronic inflammation determines disease evolution and severity beyond mere bacterial presence (27, 28). In this inflammatory environment, viruses may act as cofactors by modulating immune activity or altering local cytokine balance (29). Torque teno virus (TTV), known to replicate in immune cells, could indirectly influence disease severity through immune activation or by reflecting host immune dysregulation; accordingly, TTV might function either as an amplifier of inflammation or as a biomarker of its intensity rather than a direct causal agent. This dual perspective supports the need for longitudinal and mechanistic studies to clarify whether TTV contributes to or merely mirrors periodontal inflammation (30).

The contemporary perspective positions microbial dysbiosis as the central axis of periodontal pathogenesis, creating a dynamic inflammatory microenvironment in which non-bacterial pathogens, such as viruses and fungi, together with host-modulating factors, act as cofactors or enhancers of the immune response, influencing disease severity and progression (30, 31). Within this conceptual framework, our observations align with current literature. Hajishengallis proposes that pathogenesis is not limited to bacterial presence, but that dysbiosis generates an environment where viruses and the host response play modulatory roles, particularly in microbe–host interactions and chronic inflammation (32). Complementarily, Kinane emphasizes the central role of the host and persistent inflammation, suggesting that non-bacterial factors can modify the inflammatory response and, consequently, disease severity (23). Several recent studies have explored the role of viruses in periodontitis using viral metagenomic approaches and molecular techniques. In China, Zhang et al. (18, 20) reported that TTMV-222 has higher prevalence in patients with periodontitis than in healthy individuals, supporting the hypothesis that specific viral variants may be associated with periodontal inflammation and clinical severity (18, 20). Whole-genome and ORF1 analyses revealed notable divergences among strains, suggesting the possible existence of new Torque Teno minivirus species. Similarly, TTMV prevalence varies widely across geographic regions: studies in Uruguay (33), Romania (34), Qatar (35), Iran (36), and Taiwan (37) report rates ranging from 5% to 90%, which may relate to ethnic and environmental factors, highlighting the need for further research to clarify population distribution and its association with chronic periodontitis.

Although two of the included studies investigated TTMV and three investigated TTV, both viruses are considered members of the same Anelloviridae family, sharing similar biological behavior and tissue tropism. The main distinction between them is genome size, with TTMV possessing a shorter circular DNA genome, which does not currently imply functional or pathogenic differences. For this reason, both subtypes were analyzed collectively, as their inclusion reflects the broader anellovirus prevalence in periodontal environments (38).

Detection of TTV by PCR and metagenomics underscores the value of these tools for identifying emerging pathogens in the periodontal environment, raising questions about their biological role in pathogenesis (39). Studies by Yu, Tian et al., using gingival epithelium and periodontal tissue samples, corroborate the association between TTMV-222 and periodontitis, reinforcing the idea that the periodontal virome may contribute significant variability to local inflammation. Similarly, TTV has been associated with both chronic and aggressive periodontitis, suggesting that viral load may reflect or contribute to the inflammatory burden in different disease forms (22). Priyanka et al. (21) reported detection of TTV in subgingival and atherosclerotic plaque, suggesting possible links between oral and systemic processes.

Our review strategy was designed following international recommendations for systematic reviews and meta-analyses, considering oral samples (saliva, gingival crevicular fluid, subgingival plaque) and various detection techniques (PCR, qPCR, metagenomics). The search was conducted across multiple databases (PubMed/Medline, EMBASE, Web of Science, Scopus, Cochrane, and regional databases) with dual screening and resolution of discrepancies by a third reviewer, in accordance with methodological guidelines to reduce bias and ensure transparency. Inclusion of cross-sectional, cohort, and case-control studies allowed structured comparisons across populations and methods, providing a basis to estimate viral burden associated with the disease. However, variability in detection methods, sample types, and clinical definitions of periodontitis introduces heterogeneity and potential measurement bias. This observation aligns with previous literature noting geographic and methodological differences affecting prevalence estimates (33–37). Therefore, results should be interpreted cautiously, considering sensitivity and subgroup analyses.

Our findings show a significant association between TTV and periodontitis: four observational studies, including 300 patients and 283 controls, revealed a Mantel-Haenszel random-effects model RR of 1.67 (95% CI: 1.28–2.17; *p* = 0.00013). Heterogeneity was low (*I*^2^ = 0%), indicating consistency in effect direction and magnitude. These results are consistent with previous studies that identified TTV/TTMV in oral samples from individuals with and without periodontitis, supporting the hypothesis that viruses act as cofactors modulating local inflammation (22, 38, 39).

Nevertheless, available evidence is mostly observational, limiting causal inference. Geographic and methodological variability underscores the need to standardize diagnostic criteria, sample types, and detection thresholds in future research. TTV presence may reflect prior viral exposure or co-infections rather than a direct causal effect, highlighting the necessity of longitudinal studies to evaluate infection temporality, quantify viral load, and explore interactions with the microbiome and host immune response.

Understanding TTV's role in periodontitis opens opportunities for risk stratification, development of complementary biomarkers, and therapeutic strategies aimed at modulating local inflammation, consolidating a comprehensive approach to periodontal disease.

### 4.1 Limitations

Our study has certain limitations that, rather than diminishing its value, strengthen interpretation and guide future research. The limited number of included studies and their mostly observational nature restrict the ability to establish a causal relationship between Torque Teno Virus (TTV) infection and periodontitis. Additionally, variability in viral detection methods (conventional PCR, qPCR, metagenomics) and sample types analyzed (saliva, gingival crevicular fluid, gingival tissue, subgingival plaque) introduces methodological heterogeneity that may influence prevalence estimates. Concentration of research in specific geographic regions limits generalizability. Finally, the absence of longitudinal studies prevents assessment of infection temporality and its role in periodontal disease progression or severity.

### 4.2 Clinical relevance

Despite these limitations, the findings have relevant clinical implications, suggesting that TTV could be considered a potential complementary biomarker in the diagnosis and monitoring of periodontitis. Viral detection in periodontal tissues and fluids raises the possibility that the virome modulates the inflammatory response, which may explain variations in disease severity and progression among individuals with similar bacterial profiles. If future studies confirm this association, integrating viral detection into periodontal clinical practice could improve risk stratification, support more personalized therapies, and enable early identification of patients at higher risk for disease progression.

## 5 Conclusion

Torque Teno Virus (TTV) is more frequently detected in patients with periodontitis, suggesting a potential role in disease development. Although a causal relationship cannot yet be confirmed, this finding opens new avenues for better understanding periodontitis and exploring its value as a biomarker in clinical practice.

## Data Availability

The raw data supporting the conclusions of this article will be made available by the authors, without undue reservation.
